# Multipolar scattering and collective mode engineering in SiO₂@C core–shell nanoparticles and clusters

**DOI:** 10.1038/s41598-025-31142-7

**Published:** 2025-12-05

**Authors:** Baseerat Bibi, Yuhan Jiang, Asim Mumtaz, Luwei Yang, Linlin Guan, Fang Zhao, Lingmu Zeng, Youtong Liu, Zhu Liu

**Affiliations:** 1 School of Physics and Astronomy, Yunnan University, Kunming, 650504 People’s Republic of China; 2 School of Physics, Electrical and Energy Engineering, Chuxiong Normal University, Chuxiong, People’s Republic of China; 3 School of Physics, Engineering & Technology, University of York, Heslington, York, YO10 5DD UK

**Keywords:** Nanoparticles, SiO_2_@C nanostructures, Finite element method, Optical scattering, Magnetic quadrupole modes, Nanoparticle clusters, Materials science, Nanoscience and technology, Optics and photonics, Physics

## Abstract

The scattering properties of nanoparticles are crucial in applications like optical sensing, photonics, and medical diagnostics. Despite extensive research, the interaction of multipolar resonances in metal-free nanophotonic structures remains underexplored. This study investigates the effects SiO_2_ nanoparticle size, carbon shell thickness, and cluster topology on the excitation and evolution of multipolar resonances. We used the finite-element method in COMSOL Multiphysics to investigate the scattering of SiO₂@C core–shell nanoparticles and their clusters, validating the simulations against Mie theory. Optimal performance was observed in SiO_2_ nanoparticles with a 200 nm radius, where multipolar resonances were most pronounced, achieving a balance between enhanced scattering intensity and well-defined resonance peaks. The carbon shell thickness, varied from 5 to 100 nm, induced significant spectral shifts and intensity modulation, particularly enhancing the electric dipole (ED), magnetic dipole (MD), and electric quadrupole (EQ) modes. Notably, the magnetic quadrupole (MQ) mode emerged as the dominant contributor to scattering, underlining the crucial role of shell thickness in controlling optical properties. For nanoparticle clusters ranging from dimers to heptamers, the MQ mode remains the dominant contributor, with sharp oscillation peaks intensifying as cluster size increases. However, the introduction of a central nanoparticle in pentamer and heptamer clusters leads to reduced scattering intensity due to destructive interference and disrupted phase coherence. These findings establish the governing influence of particle arrangement and symmetry on scattering in SiO₂@C core–shell nanoparticle clusters, providing a robust framework for designing low-loss, spectrally tunable nanophotonic structures for dielectric metasurfaces and refractive-index sensing.

## Introduction

Numerical simulations are essential tools in nanophotonics to examine the optical properties in complex nanostructures without requiring fabrication or experimental measurement. Combined with optimization techniques, these simulations explore and guide structure design with enhanced photonic performance by systematically navigating high-dimensional parameter spaces to identify optimal configurations that account for linear and nonlinear optical responses^1–3^. Modern computational frameworks extend this capability further by modeling nanoscale phenomena involving molecular dynamics and quantum–mechanical effects^4–7^. These simulation-driven insights provide a foundation for the broader field of nanophotonics, where control over electromagnetic fields at the nanoscale is leveraged to achieve applications in sensing, energy harvesting, and next-generation photonic devices^8^. Within this context, core–shell nanoparticles have attracted particular interest due to their exceptional tunability, which can be achieved by adjusting core radius, shell thickness, and constituent materials. Structural tunability has made core–shell nanostructures valuable across diverse electronics, optics, catalysis, and biomedicine applications^9^.

Nanoplasmonics, a critical sub-field of nanophotonics, has been revolutionized by metallic nanostructures. The collective oscillation of free electrons at the metal surface, called surface plasmon resonance (SPR), allows metals such as gold and silver to enable enhanced electromagnetic field confinement, leading to intense scattering and absorbing incident light at nanoscales^10,11^. Such metal-based systems have proven critical in surface-enhanced raman spectroscopy (SERS)^12^, light harvesting^13^, and plasmon-driven catalysis^14^. However, the inherent optical losses and thermal instability of metallic nanostructures limit their performance for low-loss photonic applications. To overcome these drawbacks, doped semiconductor nanostructures, such as WO₂ and n-type Si, have recently been explored for plasmonic-like properties, where free carriers introduced through doping enable tunable plasmonic resonances similar to metals^15–17^. Recent advances in near- and far-field optical measurements have enabled direct observation of multipolar resonances in dielectric and hybrid nanostructures^18^. Plasmonic behavior in semiconductors arises only when free carriers are introduced through doping, as in ITO or n-doped Ge^19–21^. Moreover, metal@dielectric nanostructures demonstrate both electric and magnetic dipole resonances through plasmon–dielectric coupling, effectively linking plasmonic and dielectric regimes^22,23^. Despite the progress of metallic and semiconductor plasmonics, growing interest has turned toward all-dielectric or hybrid dielectric–conductive nanostructures, which combine strong scattering with reduced absorption loss. Dielectric materials like SiO₂ have attracted attention due to their low-loss and high-stability properties, making them ideal for controlling the scattering of light without dissipative losses associated with metal^24^. All-dielectric nanostructures can enable multipolar resonance modes like ED, MD, EQ, and MQ, expanding their application scope^25,26^.

Silica (SiO₂)-based core–shell nanoparticles are exceptionally adaptable, with applications extending from microwave absorption^27^ and random lasers^28^ to plasmonic devices^29^. Morphology and interfacial design critically influence their optical behavior. For instance, Machado et al.^28^ showed that crystalline SiO₂ nanoparticles with sharp edges are more effective in scattering efficiency than their amorphous counterparts due to electromagnetic field localization at sharp edges. Similarly, Ding et al.^29^ and Qin et al.^30^ showed that shell material and geometry could control localized surface plasmon resonance (LSPR) and even assist magnetic resonances in Ag@SiO₂ and Ag–SiO₂–Ag systems. Al-Qadi’s^9^ FEM simulations on SiO₂@Au nanoshells also underlined the significance of multipolar resonances (quadrupole, octupole) in larger nanoshells and revealed tunable plasmonic responses with shell thickness controlling blueshift-redshift crossover. Nayfeh et al.^31^ further showed that metal–silicon core–shell nanostructures produce tunable plasmon–polarizmon resonances and hotspots at both metal–semiconductor and semiconductor–vacuum interfaces, offering moderated field enhancements valuable for Raman and Luminescence applications.

Despite these advancements, hybrid dielectric–carbon systems such as Silica@Carbon (SiO₂@C) remain comparatively underexplored. Unlike metal or purely dielectric nanostructures, these hybrids offer a distinctive combination of broadband absorption with conductive damping from the carbon shell and strong dielectric scattering from the SiO₂ core. Recent studies on FAPbI₃/Au@SiO₂ bilayers^32^ and Cu@C^33^ demonstrates how these combinations can enhance exciton–plasmon coupling, field confinement, and absorption. Xu et al.^34^ demonstrated that the size of Cu@C core–shell nanoparticles profoundly affects their optical properties, emphasizing the importance of precise control over nanoparticle size in optimizing scattering behavior.

Moreover, multiparticle interactions are essential in modulating scattering and resonance behaviors. According to Shi et al.^35^, dimers of silicon nanoparticles on gold substrates show strong electromagnetic field confinement due to coupled electric and MD modes, which results in octupole modes and Fano-like resonances. Saini et al.^36^ systematically determined electromagnetic hotspots in gold nanoparticle dimers and trimers through controlled interparticle spacing. Recent computational and experimental investigations further highlight the significance of cluster geometry and coupling in determining plasmonic responses. Finite element simulation reveals pyramid-shaped gold nanoparticle dimers having nanometre-scale gaps to exhibit substantial localized field enhancements due to bonding dipole plasmon coupling in hotspots regions^37^. Self-assembled gold nanoshell trimers support MD resonances, confirmed through polarization-resolved scattering measurements, indicating complex multipolar mode formation^38^. Moreover, silver nanosphere septamers exhibit narrow Fano resonances resulting from interference between superradiant bright and subradiant dark plasmon modes, tunable by cluster symmetry and spacing, with implications for enhanced sensor response and spectral selectivity^39^. These results highlight the importance of geometry, inter-particle coupling, and interference for creating clusters with tailored absorption and scattering—principles we extend to hybrid dielectric–carbon core–shell nanoparticles. Notably, most prior studies concentrate on metallic or pure dielectric systems, leaving hybrid dielectric–carbon systems largely underaddressed.

In this work, we investigate the evolution of multipolar scattering behaviors, such as the ED, MD, EQ, and MQ modes, through systematic variation of SiO₂ core radius, carbon shell thickness, and cluster configuration. Using FEM-based simulations, we identify an optimal core size, investigate how shell thickness and particle arrangement modulate resonant modes, and extend the single-particle findings to core–shell clusters from dimers to heptamers, highlighting near-field coupling and symmetry-driven effects. To ensure the physical reliability of our results, the simulations are benchmarked against Mie theory. This study establishes a geometry-driven design framework for metal-free, low-loss nanophotonic components with direct relevance to metasurface and refractive-index sensing applications.

## Methodology

### Numerical method using COMSOL multiphysics

**Fig. 1:**
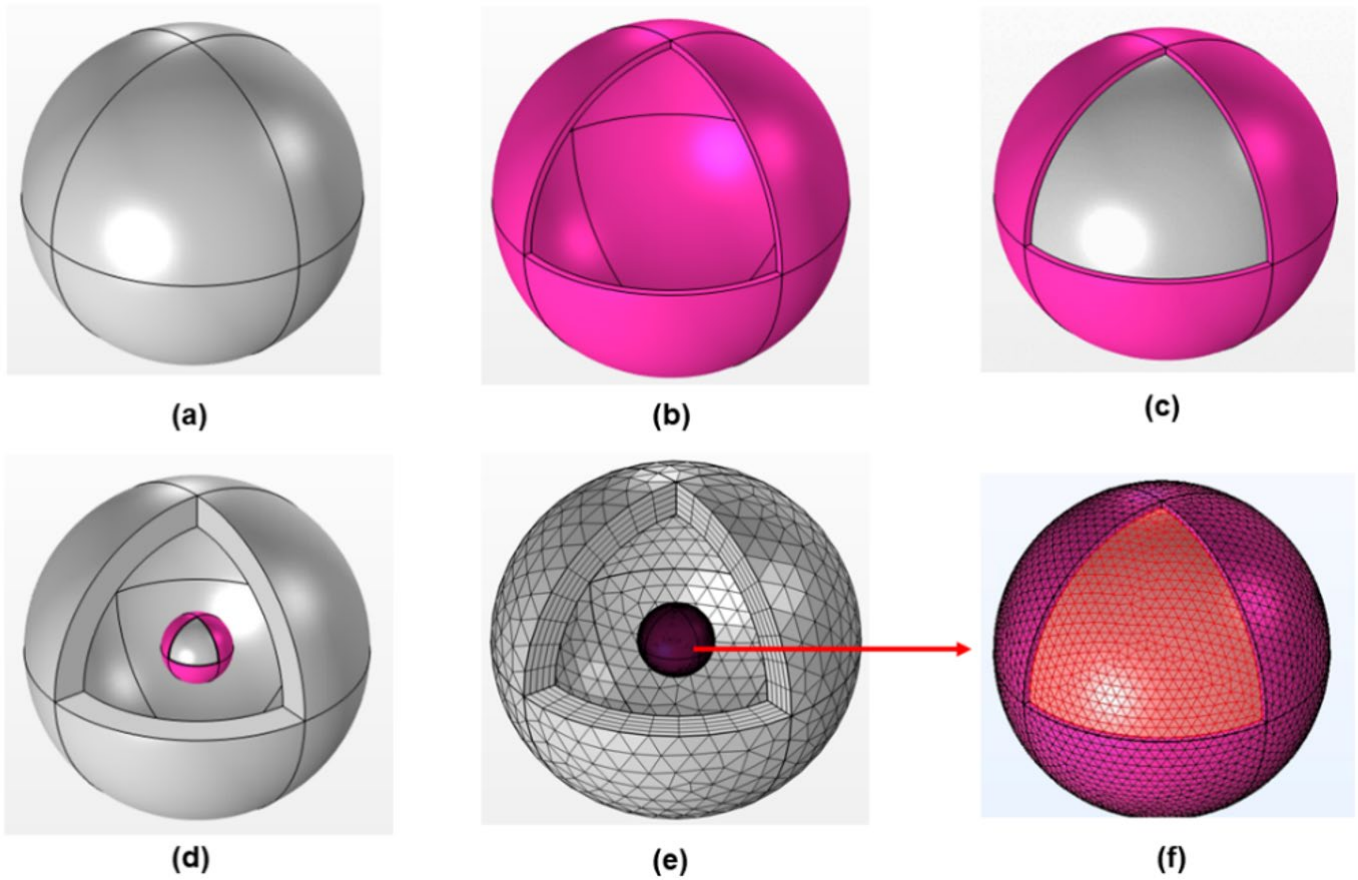
Three-dimensional model development and meshing details. (**a**) SiO_2_ core; (**b**) carbon shell; (**c**) combined core–shell SiO_2_@C structure; (**d**) Model embedded inside the PML domain; (**e**) full domain mesh; (**f**) detailed mesh within nanoparticle.

The optical scattering properties of SiO_2_@C nanoparticles were studied numerically using the FEM as implemented in COMSOL Multiphysics (version 6.1). The computational approach began with constructing a spherical nanoparticle shape centered within a spherical air-filled simulation domain, as shown in Fig. 1a-f. For the core–shell structure, a spherical SiO_2_ core with a radius (r) of 200 nm was surrounded by a concentric 10 nm thick carbon shell, all embedded in a background air medium of refractive index n = 1.0. The outer boundary of the simulation domain was enclosed by a spherical perfectly matched layer (PML) extending to five times the nanoparticle radius to absorb scattered radiation and simulate open boundary conditions, in accordance with established finite-element modeling guidelines for electromagnetic wave scattering^40,41^. A linearly polarized plane wave was used as the excitation source, with the electric field oriented along the x-axis and propagating along the + z-axis in COMSOL’s scattered field formulation.

**Fig. 2:**
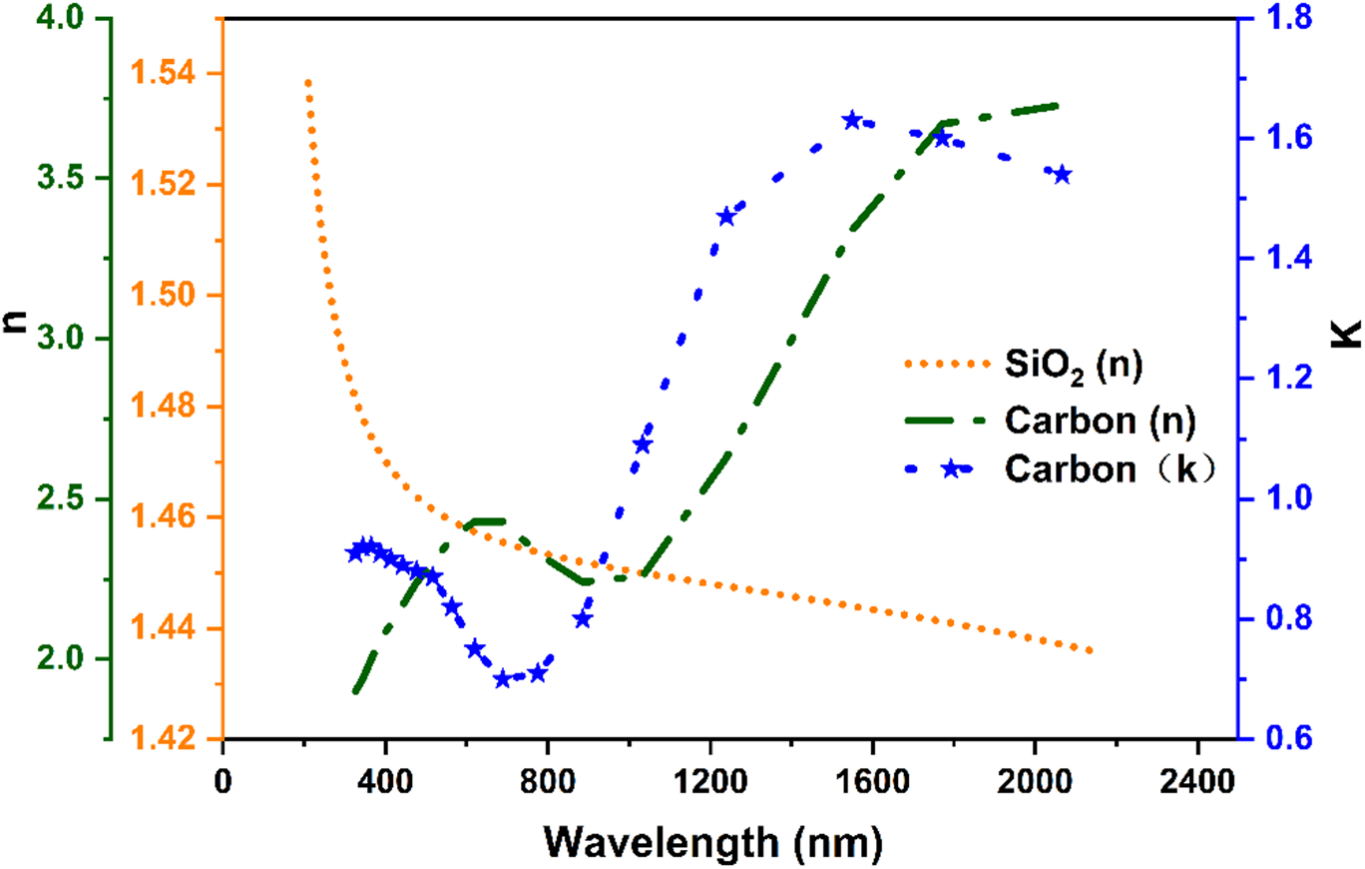
Wavelength-dependent refractive index of SiO_2_ and carbon used in the simulations.

The electromagnetic response was modeled using the wave optics module’s wavelength domain interface. The scattered field formulation was employed to isolate the nanoparticle response from the background illumination. The wavelength-dependent complex refractive indices of SiO₂ and carbon used in the simulations were implemented via interpolation functions in COMSOL Multiphysics. For SiO₂, values were obtained from tabulated experimental data^42^, and for arc-evaporated carbon, from ellipsometric measurements reported by Arakawa et al.^43^. These datasets were incorporated as real (n) and imaginary (k) components via linear interpolation. The real part of the complex refractive index, n = n + ik, is n, which represents the phase velocity of light in the material, and the imaginary part, k, or extinction coefficient, which means absorption losses. Figure 2 shows the wavelength-dependent refractive index of SiO_2_ and carbon. The accurate inclusion of both components is critical to modeling the scattering and absorption behavior of the core–shell nanoparticle. The computational method solved Maxwell’s equations in their time-harmonic form for the scattered electric field E_sca_:

1 $$\nabla \times \left[\frac{1}{{\mu }_{r}}\left(\nabla \times {E}_{sca}\right)\right]-{k}_{0}^{2}\left[{\varepsilon }_{r}-\frac{j\sigma }{\omega {\varepsilon }_{0}}\right]{E}_{sca}=0$$

where, ${\mu }_{r}$ and ${\varepsilon }_{r}$ are the relative permeability and permittivity of the medium, ${\varepsilon }_{0}$ is the vacuum permittivity, ${K}_{0}=\frac{2\pi }{\lambda }$ is the free-space wave number, and $\sigma$ is electrical conductivity. Additionally, multipole decomposition of the scattering response was performed based on the framework provided by Evlyukhin et al.^44^. Multipole moments were evaluated by integrating the induced polarization density throughout the nanoparticle volume using a COMSOL operator limited to the core–shell region. Following the standard treatment for subwavelength particles, the expansion was terminated at the quadrupole order, including ED, MD, EQ, and MQ contributions. The induced polarization density P is as follows:

2 $$P\left(r\right)={\varepsilon }_{0}\left({\varepsilon }_{p}-{\varepsilon }_{d}\right)E(r)$$

where ${\varepsilon }_{p}$ the relative permittivity of the nanoparticle, ${\varepsilon }_{d}$ is the relative permittivity surrounding medium, and $E(r)$ is the total electric field inside the particle. The mathematical expressions for the ED, MD, EQ and MQ are as follows:

3 $${ED}_{i}= \underset{V}{\int }{P}_{i}\left(r\right)dV, i=x,y,z.$$

4 $${MD}_{i}= -\frac{i\omega }{2}\underset{V}{\int }{\left(r\times P(r)\right)}_{i}dV$$

5 $${EQ}_{\alpha \beta }=3\underset{V}{\int }\left({r}_{\alpha }{P}_{\beta }+{P}_{\alpha }{r}_{\beta }\right)dV, \alpha , \beta =\text{ x},\text{y},\text{z }$$

6 $${MQ}_{\alpha \beta }=-\frac{2i\omega }{3}\underset{V}{\int }{\left[\left(r\times P\left(r\right)\right)\right]}_{\alpha }{r}_{\beta }+{r}_{\alpha }{\left[\left(r\times P\left(r\right)\right)\right]}_{\beta }dV$$

The total scattering cross section ${\sigma }_{sca}$, normalized by the incident intensity ${I}_{inc}$, can be calculated by summing contributions from the multipole moments as follows:

7 $${\sigma }_{sca}=\frac{{K}_{0}^{4}}{12\pi c{I}_{inc}}\left(\frac{{\left|ED\right|}^{2}}{{\varepsilon }_{0}^{2}{\mu }_{0}}+\frac{{\left|MD\right|}^{2}}{{\varepsilon }_{0}}\right)+\frac{{K}_{0}^{6}}{\pi c{I}_{inc}}\left(\frac{1}{1440{\varepsilon }_{0}^{2}{\mu }_{0}}{\sum_{\alpha , \beta }\left|{EQ}_{\alpha ,\beta }\right|}^{2}+\frac{1}{160{\varepsilon }_{0}}{\sum_{\alpha , \beta }\left|{MQ}_{\alpha ,\beta }\right|}^{2}\right)$$

A user-controlled meshing approach was employed to discretize the computational domain, balancing numerical accuracy with computational efficiency. The PML domains used a swept mesh of five layers to ensure a smooth transition and precise absorption, while the PML external surfaces were meshed with free triangular elements Fig. 1e. The remaining geometry regions were discretized using free tetrahedral elements with a predetermined finer mesh size throughout the entire geometry to obtain detailed features Fig. 1f. The study conducted three main computational investigations. First, single-particle analyses were performed on SiO_2_ nanoparticles with different radius (r) ranges from 25–200 nm. Electromagnetic simulations were performed for dielectric nanoparticles ranging from 25 to 250 nm in radius, using a 300 to 1000 nm wavelength range. For particles up to 200 nm, convergence was consistently achieved. However, for r = 225 nm and 250 nm, the model failed to converge when the lower limit of the wavelength range was set at 300 nm. This behavior is attributed to the increasing computational demand caused by red-shifted resonant modes and the need for increasingly fine meshing to resolve higher-order Mie modes^45,46^. To ensure convergence while capturing the dominant resonances, the wavelength range for these larger particles was adjusted to 400–1000 nm. Next, SiO_2_@C nanoparticle with a fixed 200 nm SiO_2_ core and varying carbon shell thicknesses (5–100 nm). Finally, we examined cluster simulations with a fixed 2 nm gap between them. The modeled SiO₂@C core–shell geometries and assembled clusters are experimentally realizable^47^, and comparable 2 nm interparticle separations have been experimentally demonstrated and computationally demonstrated in core–shell dimers^48^.

### Analytical method of mie scattering theory

To validate the numerical results obtained from COMSOL Multiphysics simulations, we performed the analytical calculations based on the extended Mie theory for spherical SiO_2_ nanoparticles. Mie theory offers an exact solution to Maxwell’s equations for the interaction of plane electromagnetic waves with spherical particles, and it has been widely used to model light scattering, absorption, and extinction of high symmetry in nanostructures^49^. The total scattering cross section is expressed as:

8 $${\sigma }_{sca}=\frac{2\pi }{{k}^{2}}\sum_{n=1}^{{n}_{max}}\left(2n+1\right) \left({|{a}_{n}|}^{2}+{|{b}_{n}|}^{2}\right)$$

where ${a}_{n}$ and ${b}_{n}$ are the Mie scattering coefficients representing the electric and magnetic multipole modes of order n, k is the wavevector in the surrounding medium. The Mie coefficients can have calculated from the spherical Bessel and Hankel functions according to the following relations.

9 $${a}_{n}=\frac{{m}^{2}{j}_{n}\left(mx\right){\left[x{j}_{n}\left(x\right)\right]}{\prime}-{\mu }_{1}{j}_{n}\left(x\right){\left[mx{j}_{n}\left(mx\right)\right]}{\prime} }{{m}^{2}{j}_{n}\left(mx\right){\left[x {h}_{n}^{1}\left(x\right)\right]}{\prime}-{\mu }_{1}{h}_{n}^{1}\left(x\right){\left[mx{j}_{n}\left(mx\right)\right]}{\prime}}$$

10 $${b}_{n}=\frac{{\mu }_{1}{j}_{n}\left(mx\right){\left[x{j}_{n}\left(x\right)\right]}{\prime}-{j}_{n}\left(x\right){\left[mx{j}_{n}\left(mx\right)\right]}{\prime} }{{\mu }_{1}{j}_{n}\left(mx\right){\left[x {h}_{n}^{1}\left(x\right)\right]}{\prime}-{h}_{n}^{1}\left(x\right){\left[mx{j}_{n}\left(mx\right)\right]}{\prime}}$$

where, $x=ka$ is the size parameter depending on the radius “a” of the spherical particle and m is the relative refractive index of the particle compared to the surrounding medium, defined as, $m=\frac{{n}_{sphere}}{{n}_{medium}}$^50^. The wave behaviour, both inside and outside the particle, and the boundary conditions at the sphere surface are captured in this formulation.

## Results and discussion

### Verification of COMSOL simulation

**Fig. 3:**
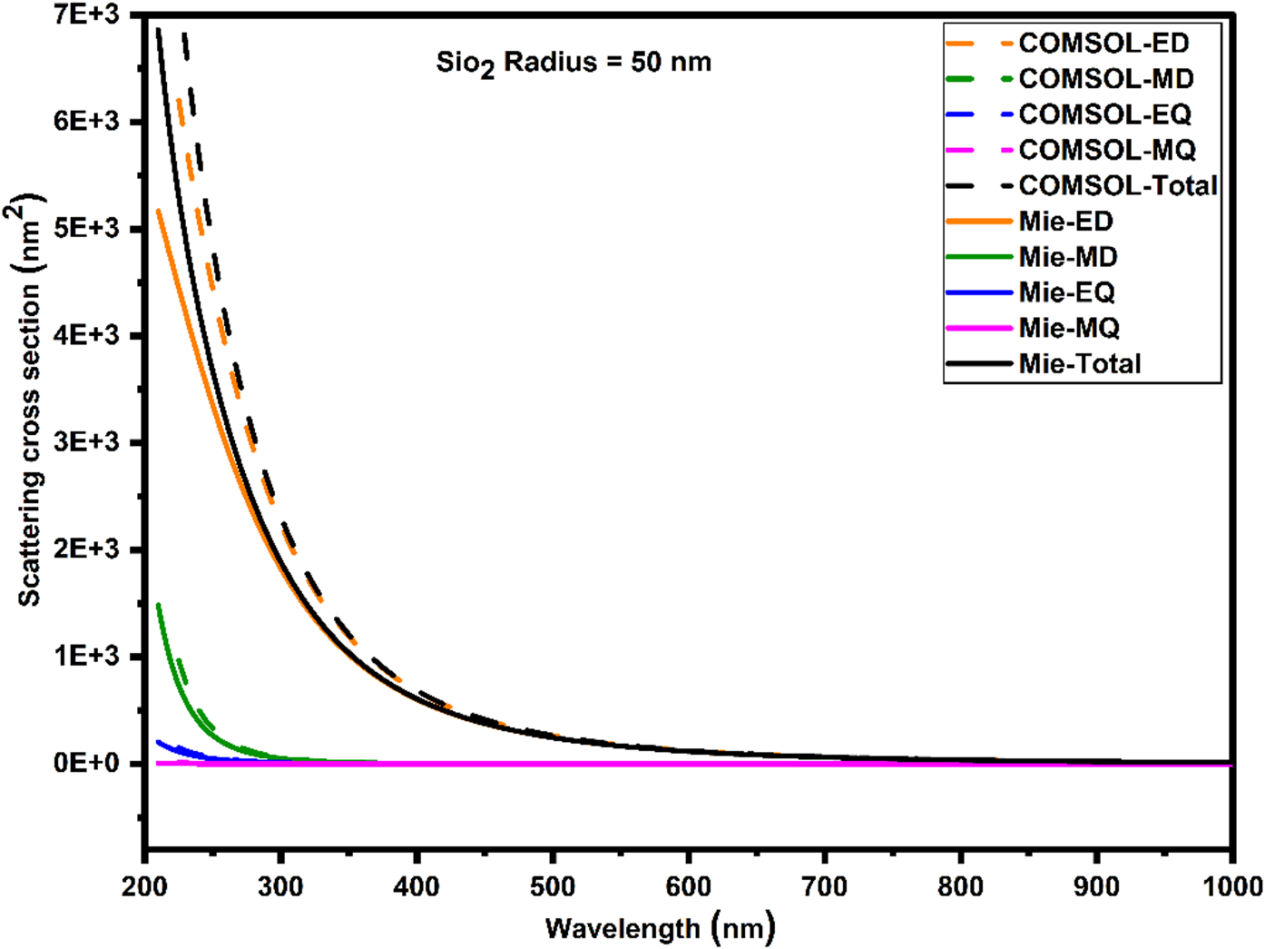
Comparison of scattering cross-section spectra for SiO₂ nanoparticle of 50 nm radius computed using classical Mie theory and COMSOL simulations.

COMSOL Multiphysics FEM simulations conducted in this study were rigorously validated by comparing the scattering cross-sections calculated from COMSOL with those obtained from classical Mie theory calculations. Both approaches were used for the 50 nm radius SiO_2_ nanospheres. Figure 3 presents the scattering cross-section spectra from both approaches, showing close agreement, particularly within spectral regions dominated by ED and MD resonances. Minor discrepancies in some spectral bands are primarily attributed to differences in boundary treatments and mesh discretization inherent to the FEM framework. The study also included multipolar scattering contributions, which were calculated from Mie theory coefficients and extracted modal components in COMSOL. The accuracy of the COMSOL FEM simulations in capturing the intricate scattering behavior of SiO_2_ nanoparticles is further confirmed by the strong correspondence between these multipolar components. This validation substantiates the COMSOL simulation approach as a reliable and accurate tool for light scattering by dielectric nanospheres.

### Size-dependent scattering cross section of SiO₂ nanoparticles

**Fig. 4:**
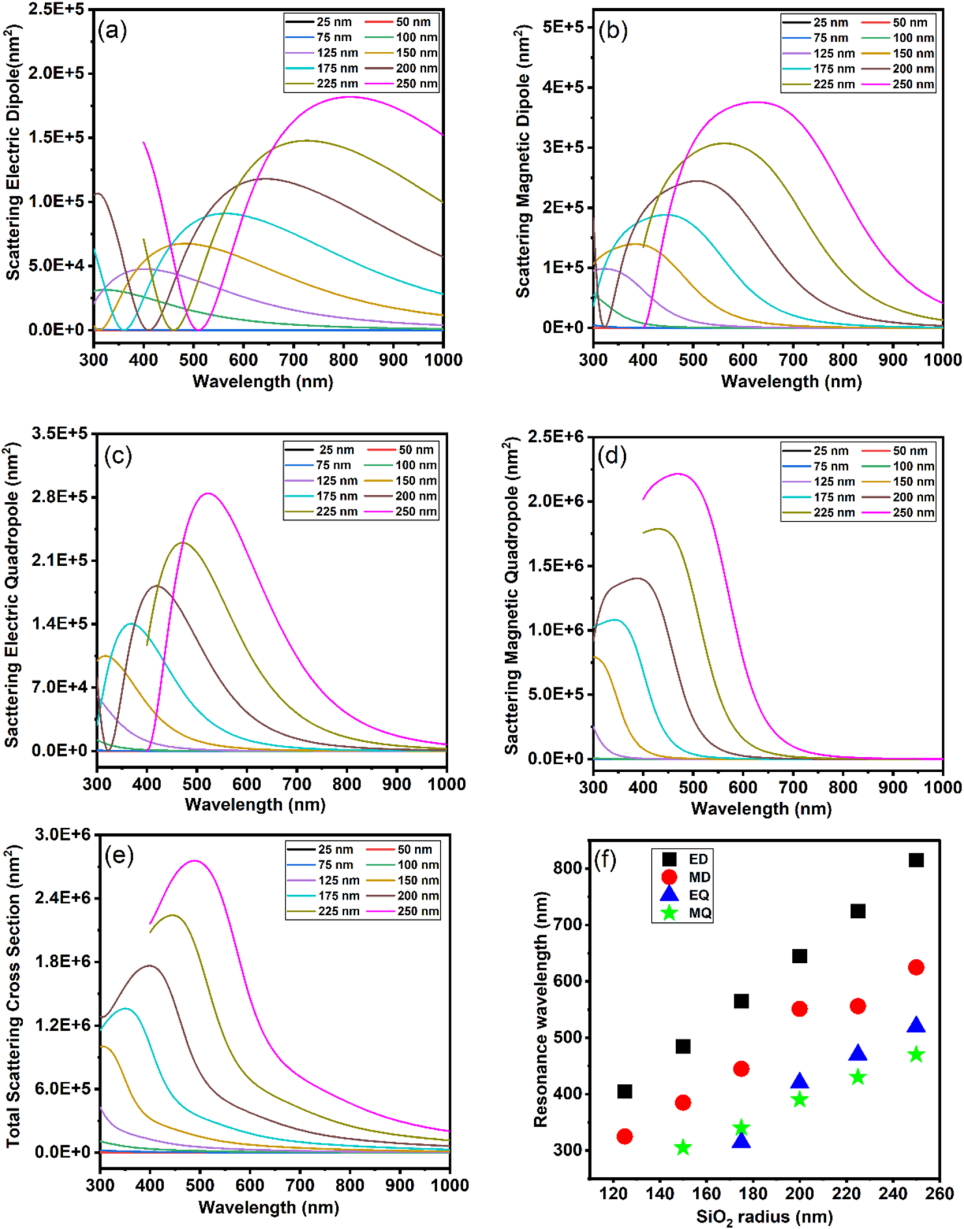
Scattering cross sections of SiO₂ nanoparticles with different radii: (**a**) Electric dipole scattering (**b**) Magnetic dipole scattering (**c**) Electric quadrupole scattering (**d**) Magnetic quadrupole scattering (**e**) Total scattering cross-section (**f**) Resonance wavelength with varying SiO_2_ nanoparticle radius.

To elucidate the size-dependent optical response of SiO_2_ nanoparticles, we systematically examined their scattering cross-section across radii ranging from 25 to 250 nm using finite-element simulations in COMSOL Multiphysics, as shown in Fig. 4a-e.

The ED scattering cross-section spectra Fig. 4a reveal distinct size-dependent trends, governed by Mie-type resonances. For smaller particles (r = 25–100 nm), the spectra show monotonic decay in intensity without distinct resonance peaks in the visible range. This behaviour is typical of Rayleigh-like scattering where dipole contributions dominate but are not resonantly enhanced. Beyond 125 nm, the resonance peak gradually redshifts from 405 nm (r = 125 nm) to 815 nm (r = 250 nm), accompanied by a threefold increase in peak scattering intensity. This redshift is attributed to retardation effects—as the particle size becomes comparable to the excitation wavelength, the phase of the incident field varies spatially across the particle, modifying the internal field distribution and delaying the re-emission of scattered waves. Additionally, larger particles exhibit broader scattering peaks, a signature of higher-order multipolar contributions, such as quadrupole and magnetic modes, that become accessible at these scales. These phenomena are well explained by generalized Mie theory and are consistent with earlier studies on multipolar interactions in dielectric nanoparticles^51^. The trends observed in our simulation also align with experimental findings where resonance shifts and intensity modulations have been linked to surface polarization and electrostatic field interactions, particularly in systems like SiO₂ where surface chemistry and ionic environments influence scattering response^52,53^. Notably, the nanoparticle r = 200 nm shows the optimal balance between spectral definition and scattering intensity in the visible light range. In contrast, the nanoparticle r = 250 nm attains the highest intensity but has a broader resonance profile, which may be undesirable for applications requiring narrowband selectivity.

Similarly, the MD scattering cross-section of SiO₂ nanoparticles shows a strong size dependence across the 25 to 250 nm radius range in the visible light range, as shown in Fig. 4b. Smaller particles (r < 150 nm) exhibit weak MD responses with no distinct resonance formation, as observed for the r = 100 nm nanoparticle, which shows a monotonically decreasing scattering profile from 300 to 580 nm. Larger particles develop pronounced MD resonances, such as the r = 150 nm nanoparticle shows a well-defined peak at 380 nm (1.4 × 10^5^ nm^2^), and the r = 200 nm nanoparticle exhibits broadband scattering from 330 to 750 nm, peaking at 510 nm (2.4 × 10^5^ nm^2^). The nanoparticle r = 250 nm displays the strongest MD response at 625 nm, having the highest intensity (3.8 × 10^5^ nm^2^), extending over a wide spectral range. This behavior arises from circulating displacement currents within the dielectric particle, which intensify when the particle size approaches the excitation wavelength, thereby enabling magnetic dipole resonance predicted by Mie theory. Wu et al.^54^ further confirmed that MD modes in dielectric metacrystals stem from such internal current loops and evolve strongly with size and geometry, consistent with modal behavior described by Mie theory. Similar size-dependent MD resonance shifts and intensity enhancements are documented in dielectric nanoparticle systems, where the magnetic resonance wavelength and strength scale with particle size and refractive index^51^. As particle size increases, this evolution illustrates the shift from simple dipole scattering to more complex scattering regimes.

Additionally, the EQ contribution becomes significant for SiO₂ nanoparticles larger than r = 125 nm, with a broad resonance peak beginning at about 300 nm and extending to about580 nm , as shown in Fig. 4c. The EQ scattering intensity of a nanoparticle r = 150 nm is 1.2 × 10^5^ nm^2^ at 400 nm, and for nanoparticles r = 200 nm, it rises to 2.0 × 10^5^ nm^2^, with a peak shifted to 500 nm. The nanoparticle r = 250 nm shows the most substantial EQ response, with resonance further redshifted to 520 nm and scattering intensity reaching 2.8 × 10^5^ nm^2^. This behavior originates from the interaction of higher-order spatial electric field distributions with the dielectric interface, resulting in longitudinal oscillations of bound charges—characteristic of quadrupole excitation. EQ modes have been shown to emerge and intensify as particle size increases and electromagnetic field confinement strengthens—particularly when the size-to-wavelength ratio supports longitudinal charge separation—highlighting their sensitivity to geometry and dielectric structure, as demonstrated in both dielectric spheres and silicon shell nanostructures^51,55^.

The MQ scattering cross-section Fig. 4d in SiO₂ nanoparticles shows a distinct evolution depending on particle size. For nanoparticles smaller than r = 150 nm, MQ scattering is weak, with scattering intensity gradually decreasing from 1.5 × 10^6^ nm^2^ at 300 nm to 5.0 × 10^5^ nm^2^ at 400 nm. More pronounced MQ resonances appear in r = 200 nm nanoparticle, which display a broad resonance centred around 390 nm with a scattering intensity of 1.4 × 10^6^ nm^2^ spanning wavelengths from 320 nm to 530 nm. The largest nanoparticle, at r = 250 nm, exhibits significantly broader resonances ranging from 350 m to 720 nm, redshifting to about 470 nm and reaching a peak scattering intensity of 2.2 × 10^6^ nm^2^. The broadening and redshift arise from the interference between MQ and ED modes, retardation effects as particle size nears the wavelength scale, and material dispersion near the UV absorption edge of SiO₂. These results are consistent with studies on dielectric nanoparticles showing that quadrupolar resonances become increasingly dominant at larger sizes, influencing scattering bandwidth and directionality^25,56^. As observed, MQ resonances begin to dominate over ED, EQ, and MD modes in the visible range, reflecting a transition toward higher-order Mie resonant behavior as particle size increases.

The total scattering cross section of SiO₂ nanoparticles exhibits a pronounced size-dependent redshift in resonance peaks as nanoparticle radius increases, as shown in Fig. 4e. Resonances shift from 350 nm at r = 175 nm to 490 nm at r = 250 nm, with an approximate redshift of 500 nm for every 25 nm increase in radius. Simultaneously, peak scattering intensity rises, accompanied by noticeable resonance broadening in nanoparticles with radii ≥ 225 nm. This evolution is attributed to increased multipolar contributions and phase retardation effects as particle size approaches the wavelength scale. These trends are consistent with earlier studies showing that size-induced spectral broadening and redshift result from higher-order resonance excitation and enhanced scattering efficiency in dielectric nanoparticles^53,57^. The results suggest that a r = 200 nm SiO₂ nanoparticle represents an optimal size, balancing scattering intensity with high spectral resolution across multiple multipolar contributions. Nanoparticles smaller than r = 200 nm tend to display weaker or less distinct resonances, whereas larger ones produce intense but broader peaks due to dominant multipolar interference. This modal transition illustrates a structure–dependent spectral tuning behavior governed by the interplay of dipolar and quadrupolar resonance dynamics.

Figure 4f shows that electric and magnetic dipoles remain active even in smaller SiO₂ nanoparticles and shift almost linearly toward longer wavelengths as the particle size increases. Quadrupole modes appear only when the radius exceeds about 150–175 nm. The overall redshift of the resonances follows the Mie size-parameter relation (x = kr)^58^, indicating that it originates from the longer optical path and phase retardation within larger particles. As the size grows, higher-order modes gradually develop alongside the dipolar ones, marking a smooth transition from simple dipole-dominated scattering to more complex multipolar Mie resonances in SiO₂ nanoparticles.

### Optical response and scattering behavior of SiO₂@C nanoparticles: the impact of shell thickness

**Fig. 5:**
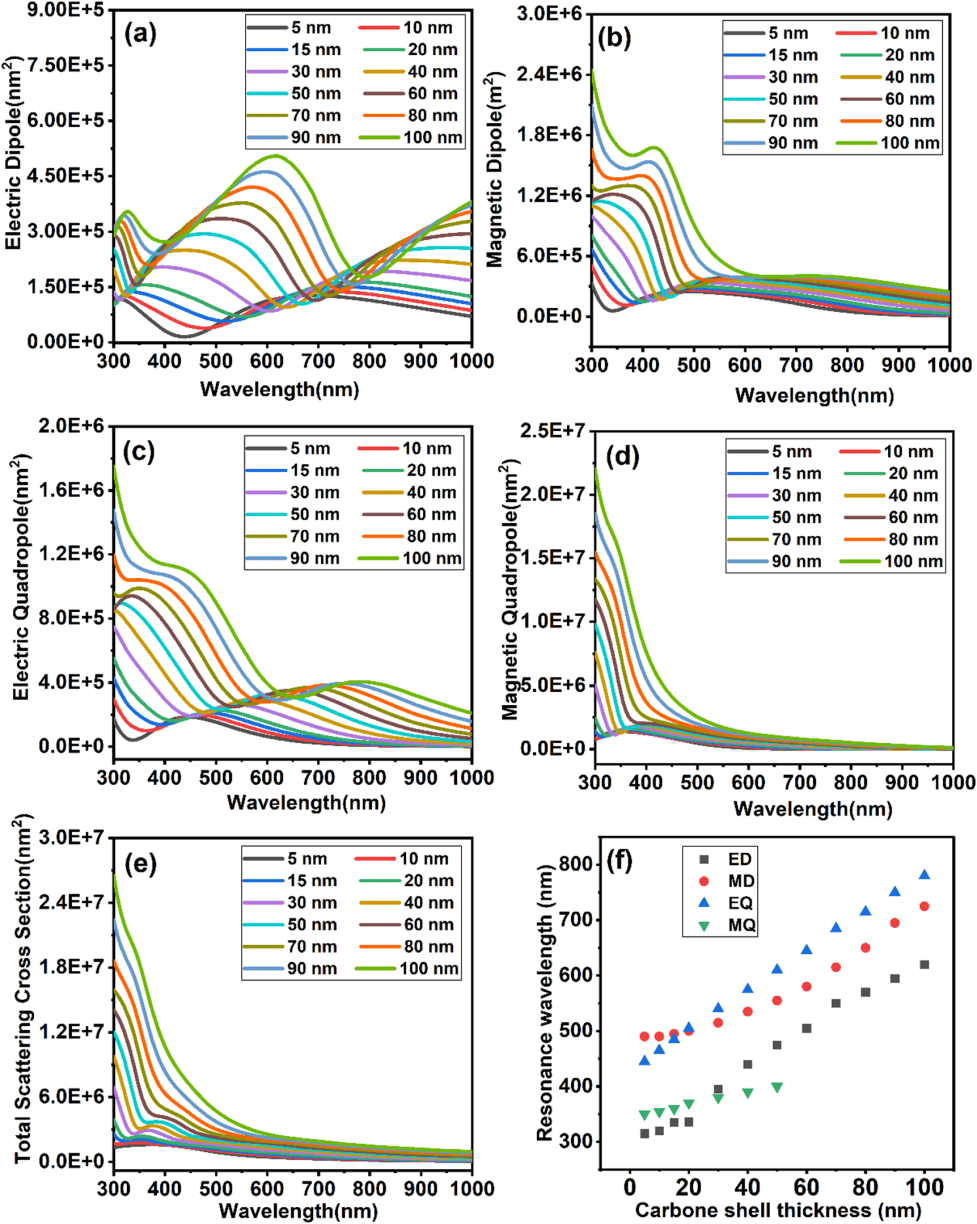
Scattering cross sections of SiO2@C nanoparticles with a fixed SiO2 core r = 200 nm and varying carbon shell thicknesses: (**a**) Electric dipole scattering, (**b**) Magnetic dipole scattering, (**c**) Electric quadrupole scattering, (**d**) Magnetic quadrupole scattering, and (**e**) Total scattering cross sections (**f**) Resonance wavelength with varying carbon shell thickness.

The optical response of SiO₂@C nanostructure with varying carbon shell thickness (5–100 nm) reveals significant changes in the scattering behaviour, as shown in Fig. 5a-e. The ED, MD, and EQ modes all redshift, and their intensities increase as the shell thickness increases. This trend is consistent with observations in SiO₂ nanoparticles, where a similar redshift and increased intensity occur with increasing particle size. However, in core–shell structures, these effects are further enhanced due to plasmon hybridization and the increase in the nanostructure’s effective refractive index, which alters the local field distribution and boundary conditions at the core–shell interface^59,60^.

In contrast, the SiO_2_@C nanostructure shows multiple distinct resonance compared to the SiO_2_ nanoparticles for all modes in the visible range. As the carbon shell thickness increases to 100 nm, the ED shows three distinct peaks: narrow features around 300 nm, a high-intensity peak near 600 nm, and a broad peak at 1000 nm. In the MD spectra, the high-intensity feature shifts below 300 nm, with a narrow peak around 400 nm and a wide feature between 600–1000 nm. Both MD and EQ exhibit similar trends. This phenomenon signifies the development of circular displacement currents and internal Mie-type loops, which align with the nanostructure’s size-dependent multipolar resonances. These resonances arise due to the complex interactions between electric and magnetic dipoles, which are enhanced as the particle size increases, consistent with the findings from nanostructure-based studies^61,62^. For the MQ, three resonances emerge as the carbon shell thickness increases to 100 nm, with the dominant peak reaching 2.0 × 10^7^ nm^2^ at approximately 300 nm, overshadowing the other two peaks. This spectral evolution results from modal interference between magnetic dipole and quadrupole modes and the activation of higher-order Mie resonances—characteristic of wavelength-scale dielectric nanostructures. Such multipolar excitations and their associated high-Q responses are consistent with all-dielectric metasurface studies, where near-field coupling and modal hybridization govern resonance shaping and spectral selectivity^63,64^. The total scattering cross-section is mainly governed by the MQ resonance across the visible spectrum, indicating a strong dependence of scattering behavior on shell thickness. This dominance arises from enhanced field confinement within the high-index carbon shell, which facilitates the excitation of higher-order Mie resonances while suppressing core–field coupling—consistent with findings from recent studies on dielectric core–shell nanostructures^58^.

Fig. 5f further substantiates these results by showing a systematic redshift of all resonance modes with increasing carbon shell thickness, owing to the enhanced refractive index and extended optical path within the carbon layer. The EQ mode redshifts most significantly up to a shell thickness of ≈ 50 nm, beyond which the resonance broadens and shifts outside the visible range, while a new feature emerges at shorter wavelengths. This indicates stronger modal dispersion and secondary resonance formation, consistent with the multipolar spectral behavior described in Fig. 5e. The magnetic quadrupole and electric dipole modes also redshift progressively, confirming that increasing shell thickness enhances interfacial polarization coupling and field confinement. Overall, the results reveal that the scattering response becomes increasingly governed by shell-induced multipolar interactions rather than by the SiO₂ core geometry.

**Fig. 6:**
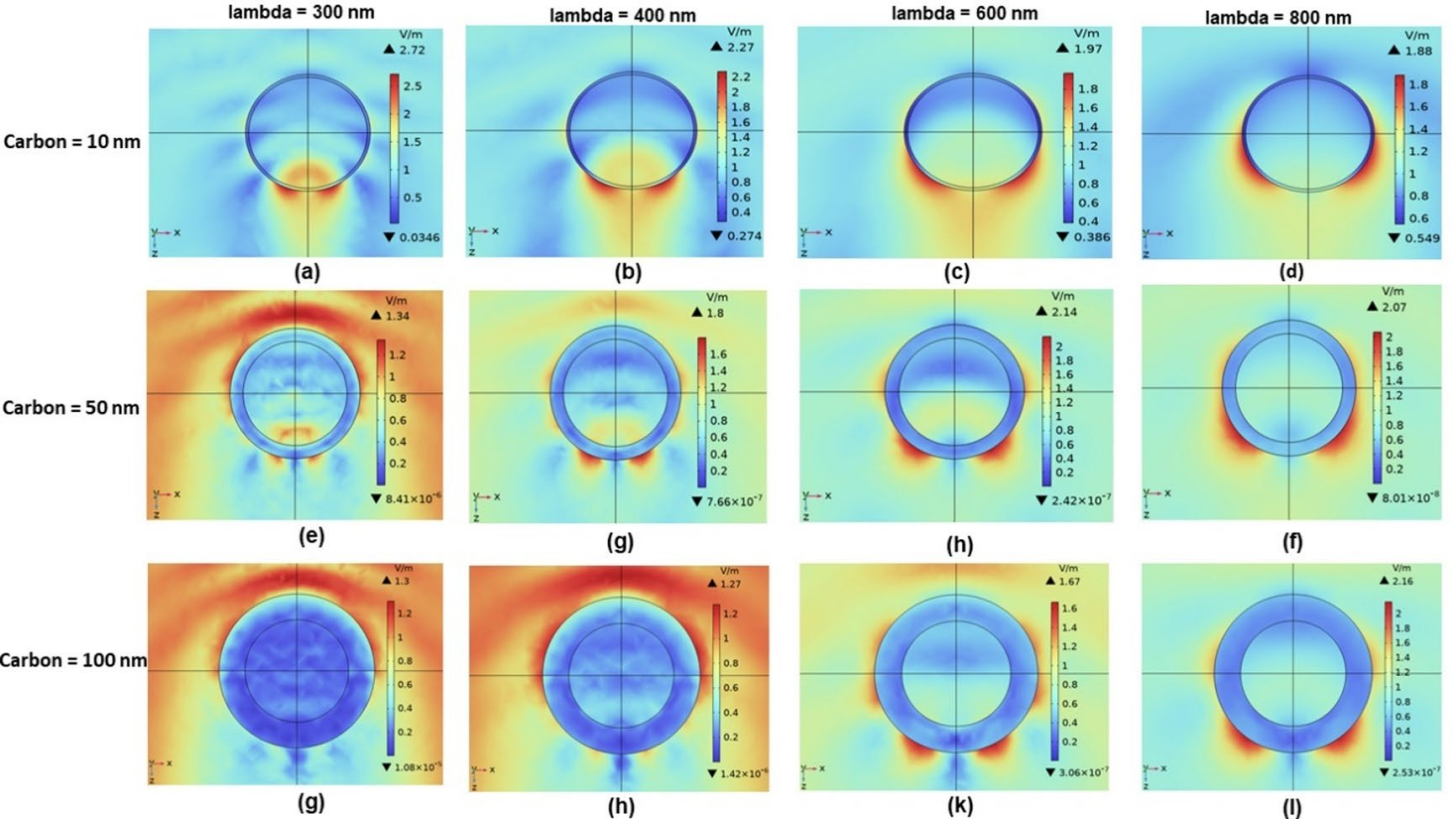
Simulated electric field distributions for SiO₂@C core–shell nanostructures with a fixed SiO_2_ core radius of 200 nm and carbon shell thicknesses of 10 nm, 50 nm, and 100 nm, under incident wavelengths of 300 nm, 400 nm, 600 nm, and 800 nm.

To gain deeper insight into the resonance behaviour observed in the scattering spectra, we conducted a detailed examination of the local electric field distributions at selected excitation wavelengths. Figure 6 presents a systematic matrix of electric field profiles for SiO₂@C nanostructures with carbon shell thicknesses of 10 nm, 50 nm, and 100 nm, under excitation wavelengths ranging from 300 to 800 nm.

The electric field for the 10 nm shell is primarily surface-localised at shorter wavelengths, exhibiting quadrupolar symmetry at 300 nm Fig. 6a and transitioning toward dipolar behaviour at 800 nm (Fig. 6d). Notably, at 600 nm and 800 nm, partial field penetration into the SiO_2_ core becomes evident, indicating continued core involvement in the resonance process. In contrast, the 50 nm shell reveals more confined field distributions. At 300 nm Fig. 6e, the field exhibits multiple lobes characteristic of octupolar behaviour. This gradually shifts toward quadrupolar symmetry at 400–600 nm Figs. 6g–h and ultimately forms a dipolar-like pattern at 800 nm Fig. 6f. While the field remains concentrated near the shell surface, its interaction with the core is notably reduced compared to the 10 nm case. For the 100 nm shell, the electric field remains completely localised within the carbon layer across all wavelengths. At 300 nm Fig. 6g, octupolar features dominate, at 400–800 nm Fig. 6h–l, the patterns maintain quadrupolar-like symmetry. Although a slight field decay toward the core is observed at longer wavelengths, the overall interaction with the SiO_2_ interior remains minimal, indicating an effective suppression of core participation in resonance modes at higher shell thicknesses.

This progression confirms that increasing shell thickness suppresses core-field interaction and enhances surface confinement while inducing a redshift in the resonant modes. Despite being visible at all thicknesses, quadrupolar features are less accessible for modal coupling and more spatially confined in thicker shells. On the other hand, thinner shells show partial core penetration and stronger field intensities, especially at longer wavelengths, which are essential for enabling hybridised resonances and near-field interactions in clustered configurations. Because of these features, the 10 nm shell configuration is especially well-suited for the subsequent investigation of SiO₂@C nanoparticle clusters, where efficient core participation and near-field accessibility are crucial for enabling inter-particle electromagnetic coupling and hybridised resonance behaviour.

### Collective scattering and modal interactions in SiO₂@C nanoparticle clusters

**Fig. 7:**
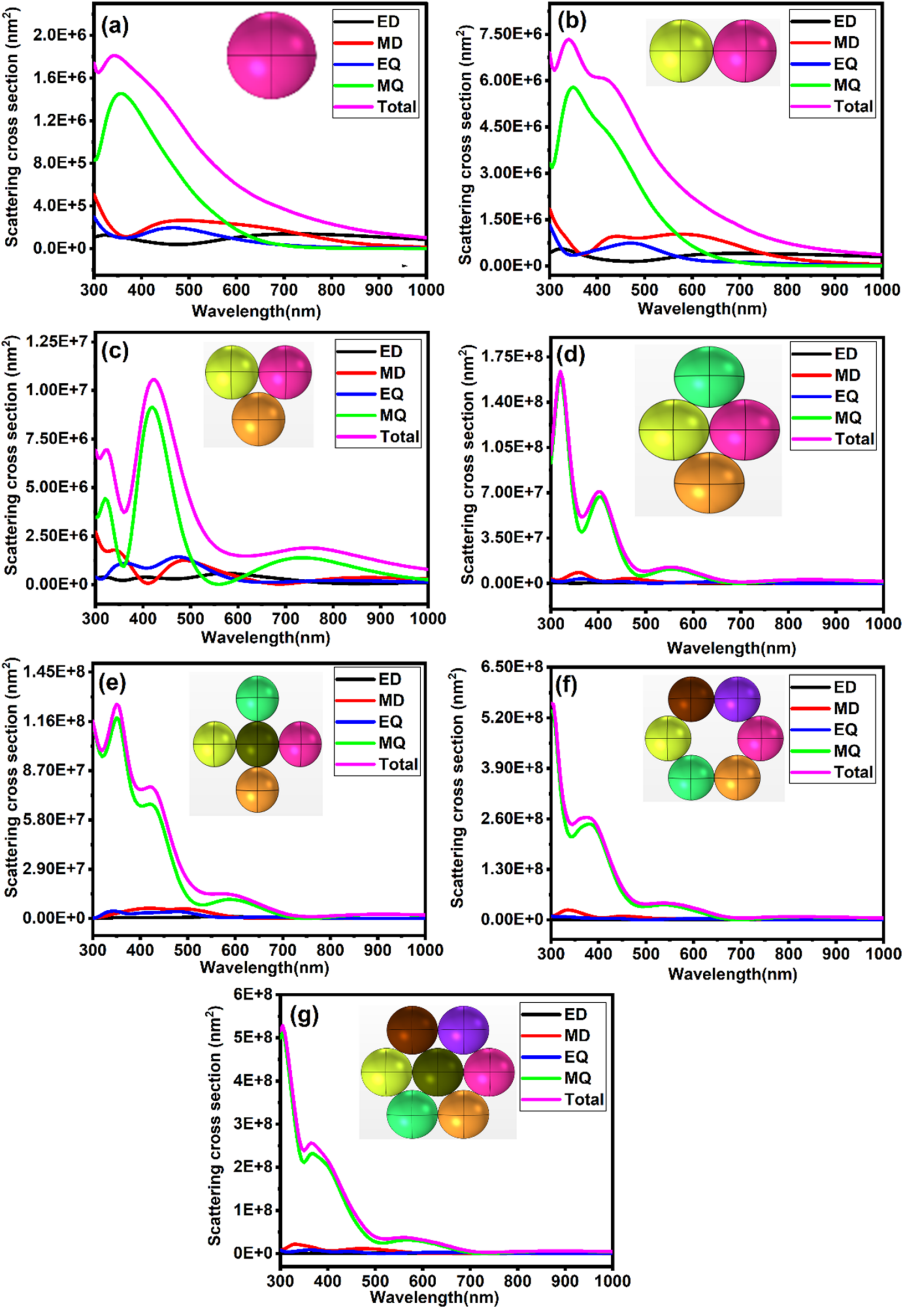
Analysis of the scattering cross section of SiO_2_@C nanoparticles: (**a**) single particle, (**b**) dimers, (**c**) trimers, (**d**) tetramers, (**e**) pentamers, (**f**) hexamers, (**g**) heptamers.

The optical response of SiO₂@C nanoparticle clusters was investigated under systematic variation of cluster topology, from dimer to heptamer, while maintaining a fixed interparticle gap of 2 nm between neighbouring carbon shells. Fig. 7a-g show scattering cross-sections from single-particle configuration to heptamer, with each nanoparticle consisting of a r = 200 nm SiO₂ core surrounded by a 10 nm carbon shell.

In the single-particle configuration, a distinct scattering peak is observed at 340 nm with a total intensity of 1.809 × 10^6^ nm^2^, mainly contributed by the MQ mode. When a second particle is introduced to form a dimer, the total scattering becomes 4 times at 340 nm, accompanied by a slight bump from the MD mode around 600 nm. With a trimer, the scattering becomes more complex, with a significant peak redshifting to ~ 400 nm and reaching 5.5 times the intensity of the single particle. The MQ mode is the primary contributor in all three configurations, whereas the ED, MD, and EQ modes become more spectrally distinct but remain comparatively weak. These findings are supported by Xu et al.^51^, who showed that resonant mode complexity rises as dielectric nanoparticles move from isolated particles to coupled systems, leading to hybridised multipolar modes and enhanced field overlap because of symmetry-driven interactions in small nanoparticle clusters.

With further increases in cluster size from tetramer to heptamer, the scattering response is completely dominated by the MQ with a sharp oscillation peak. The tetramer displays a prominent peak at 320 nm (1.636 × 10^8^ nm^2^) with the secondary resonances at 400 nm and 550 nm. Similar trends are observed in the pentamer, hexamer, and heptamer. While the ED mode shows low but consistent peaks in the 330–580 nm range (peaking at 1.19 × 10^6^ nm^2^ in the pentamer), the MD response becomes more pronounced in larger clusters, reaching up to 2.58 × 10^7^ nm^2^ in the hexamer. The EQ contribution, while spectrally broad (345–475 nm), increases slightly in intensity with particle number, ranging from 3.45 × 10^6^ nm^2^ (tetramer) to 8.55 × 10^6^ nm^2^ (heptamer). Overall scattering tends to increase with cluster size. However, this trend is not strictly monotonic, suggesting a more profound structural influence. This trend is consistent with findings by Wu et al.^65^, who demonstrated that radial arrangement in symmetric dielectric nanoparticle clusters allows for strong multipolar hybridisation and distinct mode separation, particularly between quadrupolar and toroidal responses.

A notable deviation from the expected size-dependent scattering trend occurs in the pentamer and heptamer clusters, where the inclusion of a central nanoparticle reduces total scattering intensity. Specifically, the total scattering drops from 1.636 × 10^8^ nm^2^ in the tetramer to 7.75 × 10^7^ nm^2^ in the pentamer, and from 5.5 × 10^8^ nm^2^ in the hexamer to 5.28 × 10^8^ nm^2^ in the heptamer. These observations indicate that while the central particle enhances specific coupling pathways, it also introduces destructive interference and disrupts phase coherence among radiative modes. These findings agree with Borah and Verbruggen^66^, who demonstrated that strong near-field coupling in densely packed gold nanoparticle clusters initially amplifies scattering and absorption. But beyond an optimal configuration, further inclusion of particles, especially in central or highly symmetric positions, results in modal saturation, radiative damping, and decreased intensity per particle. In this context, the central particle imposes a geometric constraint that prevents further plasmonic enhancement by breaking symmetry and redistributing the electromagnetic field. These insights highlight that strategic particle placement, not merely quantity, governs optimal optical performance.

**Fig. 8:**
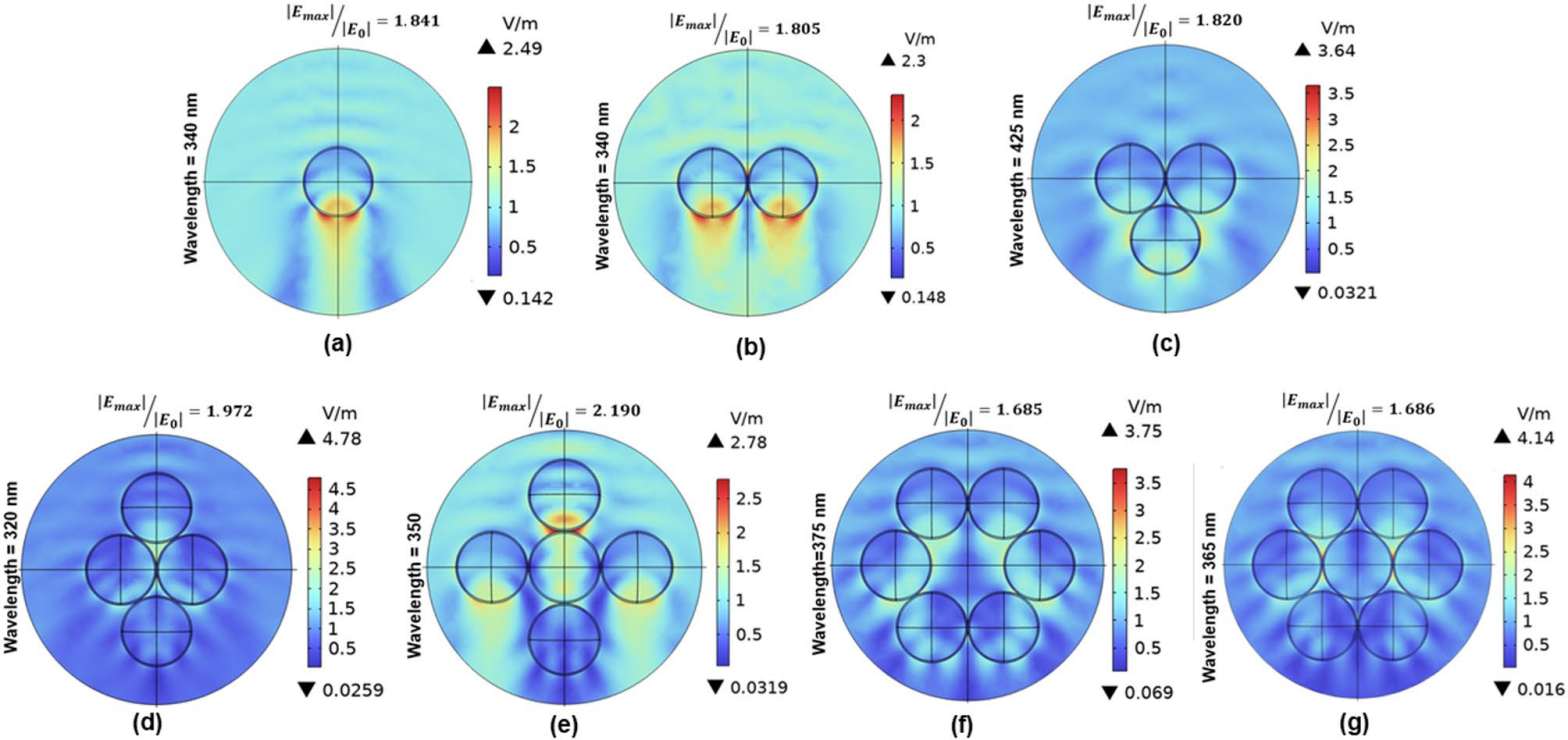
Electric field distributions at the dominant scattering resonances for various SiO_2_@C nanoparticle clusters (from single particle to heptamer) under their respective resonance wavelengths, emphasizing field variations with cluster size.

The near-field electric field distributions from single particle to heptamer configurations confirm the modal interactions inferred from spectral analysis, as shown in Fig. 8a-g. In Fig. 8, the field maps are presented using the normalized electric-field enhancement |E_max_|/|E_₀_|, which provides a direct measure of how strongly the particles localize the incident field. Interestingly, this value is highest for the pentamer and then decreases for the hexamer and heptamer. This shows that a cluster can exhibit strong local field confinement while still producing lower far-field scattering, which occurs when the added central particle introduces destructive interference and weakly radiative modes. While the single nanoparticle shows a simple dipolar field aligned with the incident light, the dimer (340 nm) exhibits significant field enhancement in the interparticle gap, indicating dipolar coupling and hybrid MQ–ED behavior. The trimer (425 nm) shows a trilobed field pattern surrounding the central void, with the most vigorous field intensities localized near the outer particles. This pattern arises from the symmetry-driven coupling between the particles, which creates a supermode where the particle arrangement causes the fields to interact coherently. In the case of a tetramer (320 nm), the field is strongly enhanced between adjacent particles, with the interparticle gaps showing the highest intensity. This results from the symmetry of the arrangement driving multipolar coupling between EQ and MQ modes, which produces hybridized MQ–EQ resonance and unique field patterns in the cluster. The field distribution in the pentamer (350 nm) shows five localized lobes, with a weaker lobe near the central particle and four around the outer particles. This pattern supports asymmetric EQ-MQ coupling by indicating a slow drop in field intensity near the center. The central particle experiences stronger coupling with particles positioned above it, resulting in more vigorous field intensity at the top. In contrast, the intensity decreases toward the bottom, leading to weaker excitation. This asymmetry arises from the particles’ geometrical arrangement and directional. At 375 nm, the hexamer displays complete radial symmetry with a confined central void, indicating coherent MQ–MD hybridization. On the other hand, the heptamer (365 nm) exhibits directional field elongation along the X-axis with minimal central excitation, indicating symmetry disruption and varied field propagation, resulting in asymmetric field distribution, decreased coupling between modes, and impaired field enhancement. These spatial features support the evolution of near-field coupling and geometry-driven coherent multipolar modes, consistent with field-mediated modal evolution described in recent research on core–shell and symmetric dielectric clusters^65,67^.

The persistent dominance of the MQ resonance across all studied configurations reveals a fundamental mechanism of energy confinement in hybrid SiO₂@C systems. The simulated spectra and field distributions consistently show that MQ excitation strengthens as size, shell thickness, and coupling increase, confirming that circulating displacement currents are efficiently confined within the carbon shell. This confinement minimizes radiative dipole losses and stabilizes high-Q magnetic resonances through geometry-induced retardation and dielectric contrast. These results establish that the MQ resonance acts as the principal channel for light–matter interaction in SiO₂@C architectures, providing a physically validated route toward achieving strong magnetic responses in all-dielectric nanostructures.

To further compare the optical performance of the SiO₂@C nanostructure, a quantitative comparison was carried out with representative hybrid systems such as SiO₂@Au^9^ and Cu@C^33^ (Table 1). Both metallic systems exhibit narrowband electric dipole resonances near 600 nm due to plasmonic oscillations but suffer from strong ohmic losses that limit their scattering efficiency. In contrast, the SiO₂@C nanostructure displays broadband multipolar Mie-type resonances extending from 300 to 800 nm, primarily governed by dielectric confinement within the carbon shell. This mechanism suppresses both radiative and dissipative losses, enhancing scattering efficiency and enabling superior spectral tunability. Overall, these results demonstrate that SiO₂@C functions as a low-loss dielectric alternative to conventional plasmonic core–shell systems, achieving stronger light–matter interactions through coupled magnetic and electric multipolar excitations.

**Table 1:** Comparison of resonance characteristics of SiO₂@C core–shell nanostructure against reported hybrid systems from the literature.

Model	Ref	Radius core nm	Shell thickness nm	Resonance wavelength nm	Mode
SiO2@Au	^9^	40	8–38	580–610	Single peak
Cu@C	^33^	5–50	2–20	600- 650	Single peak
SiO2@C	Current study	25–250	5–100	300–800	Multiple peaks

## Conclusion

This study demonstrates that the scattering properties of SiO₂@C core–shell nanoparticles are significantly influenced by the nanoparticle size, carbon shell thickness, and cluster topology, providing a flexible framework for optimizing the optical response of nanophotonic systems. The results indicate that SiO₂ nanoparticles with a 200 nm radius exhibit optimal scattering performance, with pronounced multipolar resonances and a favorable balance between high scattering intensity and well-resolved resonance peaks. Increasing the carbon shell thickness from 5 to 100 nm induced notable spectral shifts and intensity modulation, particularly enhancing the ED, MD, and EQ modes, while the MQ mode dominated the scattering behavior.

In nanoparticle clusters from dimers to heptamers, the MQ mode remained the dominant contributor to scattering, with intensity enhancing as cluster size increased. However, the introduction of a central nanoparticle in pentamer and heptamer clusters caused a reduction in scattering intensity due to destructive interference and disrupted phase coherence among the resonant modes. The near-field electric field distributions confirmed these results, highlighting the evolution of complex multipolar interactions within the clusters, driven by symmetry and particle arrangement. These findings emphasize the importance of particle arrangement and symmetry in optimizing scattering performance. These findings reveal that particle arrangement and symmetry in SiO₂@C core–shell clusters govern scattering behaviour and form the basis for designing low-loss, spectrally tunable nanophotonic structures for dielectric metasurfaces and refractive-index sensing.

## Data Availability

The datasets generated during the current study are available from the corresponding author on reasonable request.
